# Dingo Density Estimates and Movements in Equatorial Australia: Spatially Explicit Mark–Resight Models

**DOI:** 10.3390/ani10050865

**Published:** 2020-05-17

**Authors:** Vanessa Gabriele-Rivet, Julie Arsenault, Victoria J. Brookes, Peter J. S. Fleming, Charlotte Nury, Michael P. Ward

**Affiliations:** 1 Sydney School of Veterinary Science, Faculty of Science, The University of Sydney, Camden 2570, New South Wales, Australia; vbrookes@csu.edu.au (V.J.B.); michael.ward@sydney.edu.au (M.P.W.); 2 Faculté de médecine vétérinaire, Université de Montréal, Saint-Hyacinthe, Québec, ON J2S 2M2, Canada; julie.arsenault@umontreal.ca (J.A.); charlotte.nury@umontreal.ca (C.N.); 3 School of Animal and Veterinary Sciences, Faculty of Science, Charles Sturt University, Wagga 2650, New South Wales, Australia; 4 Vertebrate Pest Research Unit, New South Wales Department of Primary Industries, Orange 2800, New South Wales, Australia; peter.fleming@dpi.nsw.gov.au; 5 Ecosystem Management, School of Environmental and Rural Science, University of New England, Armidale 2351, New South Wales, Australia; 6 Institute for Agriculture and the Environment, Centre for Sustainable Agriculture Systems, University of Southern Queensland, Toowoomba 4350, Queensland, Australia

**Keywords:** *canis familiaris*, rabies, SECR, spatio-ecology, wild dogs

## Abstract

**Simple Summary:**

Australia is historically canine rabies-free but faces the threat of a rabies incursion due to the current spread of rabies through eastern Indonesia. To address this genuine concern, it is necessary to acquire further ecological knowledge on dingo populations in northern Australia to improve our predictions on the potential spread of rabies within dingoes, should an incursion occur. A one-year camera trap study was conducted in northern Australia, where the risk of the introduction of rabies is highest. Our resulting estimates of population density and home range sizes of dingoes in the study area varied according to seasons. Additionally, based on an analysis of spatial use and daily activity patterns from the camera trap pictures, a large spatial correlation and temporal overlap between dingoes and free-roaming community dogs was observed, suggesting a potential risk of disease transmission at the wild–domestic interface. This information will help improve preparedness planning for a rabies incursion in Australia.

**Abstract:**

Australia is currently free of canine rabies. Spatio-ecological knowledge about dingoes in northern Australia is currently a gap that impedes the application of disease spread models and our understanding of the potential transmission of rabies, in the event of an incursion. We therefore conducted a one-year camera trap survey to monitor a dingo population in equatorial northern Australia. The population is contiguous with remote Indigenous communities containing free-roaming dogs, which potentially interact with dingoes. Based on the camera trap data, we derived dingo density and home range size estimates using maximum-likelihood, spatially explicit, mark–resight models, described dingo movements and evaluated spatial correlation and temporal overlap in activities between dingoes and community dogs. Dingo density estimates varied from 0.135 animals/km^2^ (95% CI = 0.127–0.144) during the dry season to 0.147 animals/km^2^ (95% CI = 0.135–0.159) during the wet season. The 95% bivariate Normal home range sizes were highly variable throughout the year (7.95–29.40 km^2^). Spatial use and daily activity patterns of dingoes and free-roaming community dogs, grouped over ~3 month periods, showed substantial temporal activity overlap and spatial correlation, highlighting the potential risk of disease transmission at the wild–domestic interface in an area of biosecurity risk in equatorial northern Australia. Our results have utility for improving preparedness against a potential rabies incursion.

## 1. Introduction

Canine rabies, an acute viral zoonosis that is primarily transmitted by dogs and has the highest human case-fatality rate of any infectious disease [1], is an exotic disease of increasing concern to Australia. Canine rabies is endemic in south-east Asia and is spreading eastward through the Indonesian archipelago [2,3]. The risk of rabies entering Australia through its northern border, via the transportation of a latently rabies-infected dog on a boat from south-east Asia, is consequently increasing [4,5]. The northern Australian coastline has an extremely sparse human population over a vast area, which makes the ability of an effective surveillance of the entire coast a challenge [6]. Furthermore, the remote Indigenous communities found along the coast contain a large population of community dogs (i.e., domestic dogs that are owned by community members)—of which, a high proportion roam freely [7]. There are no resident veterinary practitioners and limited access to veterinary care services. Australia is also home to a large population of wild-living dogs (*Canis familiaris*, Linnaeus 1758), which comprises dingoes, their hybrids with modern domestic dogs and, to a lesser extent, feral modern domestic dogs [8], and these are hereafter collectively referred to as “dingoes”. The combination of these specific characteristics of northern Australia reduces the chances of early detection of a rabies incursion. Considering this context, rabies spillover from community dogs to dingo populations in northern Australia, and subsequently vice versa, would likely facilitate the spread of canine rabies on a larger scale and make disease control in Australia challenging.

Disease spread models play a role in the investigation of potential disease patterns that can inform public health decisions and strategic directions [9]. To validly represent a system of interest, the model structure should be based on knowledge of the underlying ecological and disease spread processes, with the use of accurate data for parameterisation. Population density is a key ecological parameter for disease spread models [10]; this feature has a direct impact on the potential spread of a disease within free-roaming animal populations because it can influence the contact rate between individuals [11]. Likewise, the home range size and scale of animal movement can affect the probability of contact between individuals and groups of sympatric animals through proportional spatial overlap [12]. The outputs of a disease spread model would therefore be compromised without knowledge on movement patterns, home range sizes and density estimates of the population of interest.

Dingoes are ubiquitous across most of mainland Australia, inhabiting various types of habitat ranging from tropical forests to arid regions [13]. However, the density of dingoes is likely to vary greatly across these diverse environmental zones according to their carrying capacity. In particular, dingo populations exploiting habitats neighboring human settlements might reach higher densities because of the availability of abundant and rich anthropogenic food [14]. Additionally, dingo density most likely varies temporally across seasons according to the annual dingo reproductive cycle and mortality rates [15,16]. Dingo home range sizes and movements also vary greatly between habitats and climates, often being inversely related to food and water availability [17,18,19]. For example, dingoes living near anthropogenic food resources occupy smaller areas, although some dingoes travel long distances on a regular basis to exploit these focal resources [14]. Home range sizes also fluctuate temporally with seasonal conditions and reproductive patterns [20]. These variations in dingo density and home range size cause heterogeneous encounter rates in space and time that could either facilitate or impede the spread of diseases, including rabies, in a dingo population.

Despite increasing research activity focused on dingoes, current quantitative information on dingo density is a major knowledge gap [13]. Most studies on dingo ecology report indices of activity or abundance [13], which correspond to relative measures of activity or population size that can allow the monitoring of relative population fluctuations over time and space, but do not translate to the actual number of individuals per unit area [21]. There have been no published estimates of dingo population density in equatorial and tropical climate zones of northern Australia, where the risk of canine rabies incursion is highest [13]. In fact, the only spatial transmission model of the spread of rabies among north Australian dingo populations [22] identified density as an influential parameter for determining whether a canine rabies epidemic will occur and its extent. The acquisition of density estimates and knowledge about dingo movements in northern Australia is therefore critical to improve preparedness against a potential rabies incursion.

The use of spatially explicit capture–recapture (SECR) methods is an increasingly common approach in camera trap surveys for estimating wildlife population density. These models have the benefit of overcoming the edge effect of the effective trapping area, which is inherently problematic within conventional capture–recapture approaches [23]. Spatially explicit mark–resight (SEMR) models—an extension from SECR methods—allow the estimation of population densities for which only a subset of the animals can be uniquely recognised by either artificial tagging or identification of natural marks [24], e.g., pumas (*Puma concolor*) [25], European red foxes (*Vulpes vulpes*) [26], feral cats (*Felis catus*) [27] and Canada geese (*Branta canadensis*) [28].

The objectives of our study were to: 1. generate, using a SEMR approach within a camera trap sampling design, reliable dingo density estimates; 2. estimate dingo home range areas; 3. spatially describe dingo movement patterns; and 4. investigate temporal activity overlap and spatial correlation between dingoes and community dogs in the region. The overall aim was to obtain parameters for use in spatial rabies transmission models for equatorial northern Australia.

## 2. Materials and Methods

### 2.1. Study Area

We conducted our study in the remote Northern Peninsula Area region (NPA: −10°53′16″ S, 142°23′16″ E) on the northwestern coast of Cape York, Queensland, Australia. The NPA comprises five Indigenous communities (Bamaga, Injinoo, New Mapoon, Umagico and Seisia), with small populations of people (260 [Seisia]–1164 [Bamaga] residents) [29]. The communities are surrounded by wilderness that includes tropical forests, mangroves, grasslands and wetlands [30]. The climate is equatorial, characterised by high average daily temperatures (25.5–37.9 °C), with a dry season (May–October: minimum mean monthly precipitation ~0 mm) and a wet season (November–April: maximum mean monthly precipitation >600 mm) [31]. To our knowledge, no lethal control of dingo populations occurred in the NPA during our study or previously.

### 2.2. Camera Trap Sampling Design

**Figure 1:**
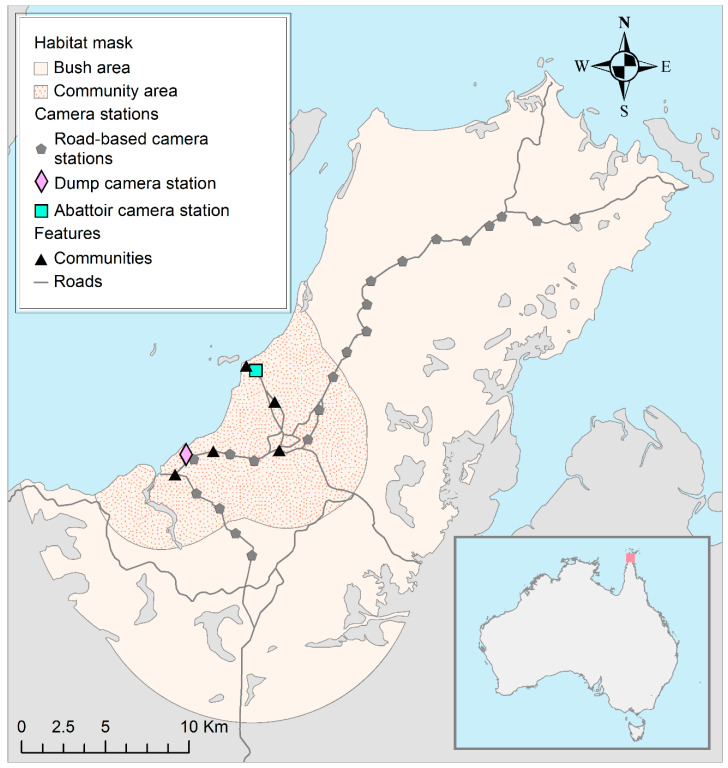
Location of the field site in the Northern Peninsula Area (NPA) of Queensland, Australia, and distribution of 23 camera trap stations operated from May 2016 to May 2017 and the habitat mask used in spatially explicit mark–resight (SEMR) models for estimating the population density of dingoes in the area.

In May 2016, we systematically deployed one camera at each of the 21 road-based stations (Figure 1) because dingoes preferentially use roads, trails or animal pads for travelling [32,33]. All camera traps were placed beside these features, mostly on small trails or pads a short distance from the main roads to avoid disturbance by humans and ensure independence between camera stations (i.e., an animal that uses the main road as a travel corridor would not be captured sequentially on multiple cameras). To enable the recapturing of individuals at different camera traps while covering a large spatial extent of the study area [34], camera trap stations were placed 2 km apart based on the smallest recorded mean home range estimate (assuming a circular home range) from similar environments to the NPA (11.2 km^2^) [13,35].

Seven additional camera traps were deployed at two focal point stations—three cameras at the refuse dump and four cameras at the local abattoir (Figure 1). These sites represented ecologically optimal focal points that, based on work in other regions [36], preliminary local studies and anecdote [37], were likely to contain the most abundant human-provided food for dingoes. At each of these focal point stations, the camera traps were treated as a single detector (i.e., non-independent) for density estimation because of their close proximity to one another.

We used RECONYX HC600 HyperFire camera traps (RECONYX, Holmen, WI, USA). Each camera was un-lured, mounted on a tree or a fence ~50 cm above the ground, oriented southward to avoid flaring of images by the sun, angled slightly downwards to focus on a point 5–6 m from the device and, in the case of road-based camera stations, set at 22° relative to the road’s orientation (after Meek et al. [38]). The GPS location of each camera site was recorded. We set each camera trap to operate continuously and to take 10 consecutive photographs in succession whenever triggered, with no time delay between triggers. All images were automatically stamped with a camera identification number, ambient temperature, date and time. Cameras were deployed for 372 days between 09-May-2016 and 15-May-2017 and serviced regularly at 9 day–3 month intervals.

### 2.3. Photograph Examination: Individual Recognition and Level of Camera Obstruction

Metadata tags were assigned to photographs using the image management software Exifpro Image Viewer [39]. Initially, every image from each set of 10 photographs per trigger was viewed and manually tagged to each animal species. Canid photographs were subsequently assigned as either “dingo-like” for unaccompanied free-roaming dogs exhibiting known phenotypic characteristics of dingoes [15] or “domestic-like”, which referred to recognised community dogs, those of a distinguishable domesticated breed, those with humans accompanying them or those displaying signs such as collars suggesting human ownership. Domestic-like photographs were further categorised as “supervised” when the dog was accompanied by a human (with or without a leash) or “unsupervised” otherwise. Photographs that could not be readily classified as dingo-like or domestic-like were tagged as “unsure”. Dingoes were classified as “marked” if an individual profile could be created from a combination of obvious and subtle distinguishing natural characteristics (after O’Connell et al. [40]) including gender, pelage colour and patterns, body size and shape, and oddities including injuries and scars [41]. All other dingo photographs were classified as either “unmarked” (individuals that were clearly not represented in any of the profiles from the marked group, because they lacked recognisable distinctive and subtle characteristics) or “mark status unknown” (individuals whose status could not be clearly distinguished between marked or unmarked due to blurred images, indistinct nocturnal photographs or partially captured animals). We subsequently discarded records from the “mark status unknown” category from the SEMR analyses because most images thus categorised were blurred or captured during nocturnal hours, and both marked and unmarked individuals would have equal probability of falling into that category.

One assigned researcher undertook an initial attempt to identify individual dingoes. The identification of each dingo photograph was then carefully revised by a second researcher. For every instance of disagreement about identification, the two researchers discussed and systematically reviewed individual features until a consensus was reached. Following the identification process, each set of 10 photographs per trigger from the marked group were further categorised independently by the two researchers into two levels of confidence: (1) “unambiguous” identification when photographs were conclusively linked to the correct profile, due to the recognition of one or multiple distinctive features which were particular for that individual and (2) “highly probable” identification when the rationale behind the identification included a combination of multiple subtle or less specific characteristics. In the case of disagreements regarding the level of confidence of identification, consensus was reached following discussions between both researchers. These two levels were created to explore the impact on the model outputs of including or excluding the highly probable identifications within the marked group.

Although we serviced the cameras regularly, we could not completely prevent obstruction by vegetation growth between visits, especially during the wet season, characterised by rapid and substantial vegetation growth. Obstruction varied across stations and time and could have affected the probability of detection of an animal. To account for this factor in the analyses, the amount of obstruction at each camera and during each 6 day interval (referred to as sampling occasions; see “Image handling and data manipulation” below) was rated using a percentage scale in intervals of 10% by studying the photographs from the entire dataset. When a camera did not trigger any photographs during a specific occasion, the amount of obstruction was interpolated from the closest photograph in time captured from that camera. A “degree of obstruction” matrix was created for each station and sampling occasion. For the multi-camera stations, the degree of obstruction was calculated as the average from all operational cameras during that sampling occasion at that specific station. A corresponding “degree of visibility” matrix was then calculated by subtracting the degree of obstruction from 100%.

### 2.4. Image Handling and Data Manipulation

To comply with the model assumption of a closed dingo population and to investigate seasonal differences, the data were divided into 4 sessions, each approximately corresponding to the first and second half of the dry and wet seasons, respectively (Session 1: 09 May 2016 to 18 Aug. 2016 (102 days); Session 2: 19 Aug. 2016 to 16 Nov. 2016 (90 days); Session 3: 17 Nov. 2016 to 14 Feb. 2017 (90 days); Session 4: 15 Feb. 2017 to 15 May 2017 (90 days)). The data were further aggregated into sampling occasions of six days to account for the potential varying detection probabilities across time and stations related to obstruction of the view of the camera by vegetation growth. All sessions therefore comprised 15 consecutive sampling occasions, except for the first session, which comprised 17 occasions.

We used the CamtrapR package [42] in R [43] to organise and manage all images, and to extract metadata for creating record databases used in the SEMR analyses. Temporal independence between subsequent records of the same or of non-distinguishable individuals at the same station was set to 30 min because this time difference is typically used for large mammals in camera trap studies [44]. This threshold corresponds to the minimum time difference between two consecutive captures of the same or non-distinguishable individuals, which were to be considered as two independent events. For the abattoir and refuse dump focal stations, photographs of an individual captured from one or multiple cameras at a given station within the 30 min threshold were recorded as one detection event. Detection histories of marked animals and counts of unmarked individuals at each station on each sampling occasion were then developed for each session. We created a trap layout file containing the geographic location of each station or, for each focal point station, a geographic centroid of all the camera traps at that station.

### 2.5. Spatially Explicit Mark-Resight Analyses

The density of dingoes across the NPA was estimated using maximum pseudo-likelihood SEMR analysis implemented in the SECR package of R [45,46]. All spatially explicit capture–recapture models (including SEMR models) combine the following features: (1) a state model describing the distribution of individual animal home range centroids, treated as a homogeneous (constant over space) or inhomogeneous (varying over space) Poisson point process, within a state space *S* and (2) an observation model describing the decreasing probability of detection of an individual at a trap as a function of distance (*d*) from the trap to the home range centroid (*g*(*d*)). The detection function *g*(*d*) is generally described by the baseline encounter probability, *g0*, or the baseline encounter rate, *λ*_0_, which is the probability or rate of detection provided that a trap is located directly at an animal’s home range centre; and sigma, the spatial scale parameter that determines the rate at which the probability of detection decreases with distance *d* and is related to the home range area of individuals. For SEMR models, the parameters from the detection function are estimated based on detection histories of the marked animals. These parameters are then assumed to be the same for the unmarked animals in order to estimate the density of the total population within the state space *S*. The marked animals were assumed to constitute a demographically and spatially random sample of the population found within the state space *S*, which is a valid assumption for naturally marked animals [24].

The likelihood for estimates of density is evaluated by integrating over the state space *S*, also called the habitat mask, which should be large enough so that home range centroids of animals located beyond the outer limit of this area would have a negligible chance of being detected by the traps. A habitat mask shapefile was created by delimiting the shoreline of Cape York Peninsula. Within this area, all habitats unsuitable for dingoes were removed from the shapefile, including (1) lakes, marine swamps, saline coastal flats, swamps, foreshore flats and watercourse areas [47] and (2) mangroves [48]. Additionally, the outer boundary of the habitat mask was delimited using a 10 km buffer surrounding our camera stations, that width being based on the largest 100% Minimum Convex Polygon (MCP: an overestimate of the actual area used [49]) home range area estimated for an individual dingo in the tropics (293.4 km^2^) [13,50]. Because the corresponding home range radius was 9.7 km, we assumed that dingoes seldom travel distances >10 km from their home range centroid. In the subsequent steps of the analyses, we verified that the 10 km buffer width was at least 3 times the estimated value of sigma in the density models. The resulting habitat mask covered an area of 606.9 km^2^ with a 676 m spacing between adjacent grid cell centres. All mapping was performed using ArcGIS version 10.5 (ESRI, Redlands, CA, USA).

For every step in the model selection process, multi-session SEMR models were employed, consequently implying that the dingo population was assumed to be closed with respect to demographics and movement within each session. Two detection functions commonly used in camera trap surveys were evaluated—that is, a half-Normal function,

(1) $$g\left(d\right)=g_{0}\exp\left(\frac{-d^{2}}{2\sigma^{2}}\right)$$

which uses the baseline encounter probability (*g0*) and the hazard half-Normal function,

(2) g(d)=1−exp(−λ(d))

(3) $$\lambda \left(d\right)=\lambda_{0}\exp\left(\frac{-d^{2}}{2\sigma^{2}}\right)\;$$

which uses the baseline encounter rate (*λ*_0_). Two models were fitted using each detection function, while keeping the detection parameters *g0* or *λ*_0_ and sigma constant across animals, occasions, detectors and sessions, and density constant spatially and across sessions (i.e., the null model). The selection of the detection function used for all following analyses was based on the comparison of Akaike’s Information Criterion value with small sample adjustment (AIC_c_) [51] produced by both competing models.

Since the number of camera traps varied per camera station, two different measures of varying effort between stations and sampling occasions were evaluated: the number of camera trap days, which is the sum of the number of days during which each camera within a station was operational during each occasion (i.e., all cameras within a station treated as fully independent); and the number of operational days, which is the number of days for which at least one camera was operational within a station during each occasion (i.e., all cameras within a station treated as fully dependent). Models accounted for varying effort by applying the hazard-based effort adjustment developed by Efford et al. [52]. Outputs from the two null models with different effort adjustment (measure of effort #1 and measure of effort #2) as well as the null model without adjustment for any effort were compared. The model producing the lowest AIC_c_ value was selected for subsequent analyses.

The following covariates were selected for evaluation on the parameters *g0* or *λ*_0_ and sigma of the detection probability (referred to as detection covariates): (1) a 2-class station-specific covariate allowing the distinction of road-based from focal point stations, tested on *g0* or *λ*_0_; (2) a continuous station-specific covariate comprising the average number of active cameras, based on days with at least one active camera, for each station, tested on *g0* or *λ*_0_; (3) the continuous degree of visibility covariate, taking into account obstruction due to vegetation growth at each station and each occasion, tested on *g0* or *λ*_0_; (4) a categorical session-specific covariate to explore differences between all 4 sessions, tested on σ; (5) a 2-class season-specific covariate to explore differences between the dry season (sessions 1 and 2) and the wet season (sessions 3 and 4), tested on sigma; and (6) a spatial 2-class environment covariate tested on σ. The latter permitted us to investigate differences in sigma, and hence home range sizes, between a “community area” (i.e., based on a 4 km buffer distance around the boundaries of the human populated areas of any of the five Indigenous communities, which were defined using roads and tracks as a reference point [53]) and a “bush area” (i.e., all remaining space within the study area). The following temporal and spatial covariates were also tested on density (referred to as density covariates): (1) the categorical session-specific covariate (sessions 1, 2, 3 and 4); (2) the 2-class season-specific covariate (dry versus wet seasons); and (3) the spatial 2-class environment covariate.

A multi-step selection approach was employed to identify the covariates that were included in the final model. As a first step, detection covariates (detection covariate 1 to 6) were included individually into the model, one by one, while allowing density to vary across sessions and environment (i.e., bush versus community area). The two detection covariates (on *g0*/*λ*_0_ or sigma) which produced the smallest AIC_c_ values were then included in a seventh model. Due the sparsity of our dataset, we did not include more than two detection covariates within a same model and interaction effects between detection covariates were not investigated. The detection covariates found within the model with the lowest AIC_c_ value amongst the seven models were selected for the subsequent analyses. As a second step, using the detection covariates selected previously, the best model for density was then selected by comparing AIC_c_ values for the six models formed by including all possible combinations between temporal (density covariates 1 and 2) and spatial (density covariate 3) covariates on density. When spatial covariates on density are integrated in the analysis, the state model is treated as an inhomogeneous Poisson point process. Models that did not converge were discarded. This model selection approach was followed using the entire dataset for the marked animals, comprising the two levels of confidence of identification (i.e., unambiguous and highly probable) and the subset of the marked dataset, which included only unambiguous identifications (i.e., excluding highly probable identifications). Model outputs resulting from these two datasets were compared. For all models fitted during the selection process, a simulation-based adjustment for spatial overdispersion in the unmarked sightings was performed by refitting each model using a maximum pseudo-likelihood approach [46].

In the event that the half-Normal detection function, which assumes a bivariate Normal model of space, was selected in our density models, we calculated the 95% bivariate home range area [28]. This area, which is assumed to be approximately circular in shape, can be derived from the spatial scale parameter estimates obtained from the final model selected using the formula from Royle et al. [54]:

(4) $$A_{95}=5.99\pi \sigma^{2}$$

### 2.6. Descriptive Analysis of Dingo Movement Patterns

To investigate whether the two focal points were associated with longer movements of dingoes across the study area, we extracted, based on all sightings of marked individuals from all stations (i.e., road-based and focal point stations), the observed net maximal distance travelled for each dingo. That is, the straight-line path (regardless of habitat) between the two cameras furthest apart at which the same dingo was detected. Pairs of camera stations consisted of either road and focal stations (regardless of the direction of movement) or road and road stations and were categorised as such. Based on dingoes that were detected in two camera stations or more (referred to as “moving dingoes” hereafter, versus “stationary dingoes”), a histogram was created to compare the distributions of the observed net maximal distance travelled per individual between each type of pairs of stations (road–road versus focal-road). The mean estimates for these were compared using a Welch’s *t*-test. Additionally, a map was created (ArcGIS version 10.5. ESRI, Redlands, CA, USA) by connecting camera stations based on the net maximal distance paths for each marked individual moving dingo. Connections between stations in the map were weighted according to the number of individuals for which each path represented their net maximal movement observed.

### 2.7. Analyses on Temporal Overlap and Spatial Correlation between Dingoes and Community Dogs

Using the package overlap in R [55], the temporal activity for each of the four types of dogs (i.e., dingo-like, domestic-like supervised, domestic-like unsupervised and unsure dogs) was analysed for each of the four sessions. For these analyses all detection events for each type of dog, using the 30 min independence threshold, from all camera stations (road-based and focal point stations) were pooled over space and time across each session, according to the time stamps of photographs. For unmarked and mark status unknown dingoes, as well as all domestic and unsure dogs, photographs of the same type of dog at the same station captured within the 30 min interval were considered as a single detection event, unless the animals were clearly individually distinguishable or multiple dogs were captured in a picture. A daily density curve in activity patterns was then obtained per type of dog and session. The coefficient of temporal activity overlap (i.e., the area under two overlapping density curves) was calculated between dingoes and the two types of domestic dogs; 95% confidence intervals were determined using a bootstrap method.

To investigate spatial correlation between dingoes and the two types of domestic dogs, we calculated the relative abundance index (RAI) for each dog type at each camera station and during each session. This index was calculated by dividing the number of detection events for a given dog-type at a given camera station during a given session by the total effort of that camera station during that session. The value of the denominator in this calculation was based on the measure of effort (effort #1 or effort #2) which produced the lowest AIC_c_ value in the model selection approach (see Section 2.5. *Spatially explicit mark–resight analyses*). The correlation between the RAI of each pair of dog type was evaluated with the Spearman rank correlation coefficient.

Research was conducted with approval from the Animal Ethics Committee of the University of Sydney (2016/1090).

## 3. Results

During the 12 month study period, the 28 camera traps captured 1.374 million photographs during 7684 camera trap days. There were some failures of cameras, mainly caused by bush fires and theft. Consequently, the camera trap days and the number of operational days varied across stations, ranging from 65 to 804 (mean, 334; median, 326) and 65 to 371 (mean, 304; median, 310), respectively. Dogs were identified in 32,666 photographs—of which, 56.2% were attributed to dingo-like dogs, 36.2% to domestic-like unsupervised dogs, 4.7% to domestic-like supervised dogs, and 2.9% to unsure dogs. All domestic-like supervised dogs observed, except for one, were not restrained with a leash and at least half of these supervised dog pictures were captured during a hunting trip. Individual (i.e., marked) dingoes were identified in 9960 photographs, compared to 590 and 8100 photographs of unmarked dingoes and mark status unknown dingoes, respectively. The marked dingo group comprised 66 uniquely marked dingo profiles. Following consideration of the temporal independence threshold, the dingo photographs corresponded to a total of 932 detection-events—882 of which were attributed to marked dingoes and 50 to unmarked dingoes. When only considering the unambiguous identifications, the number of detection events for marked dingoes decreased to 508. Throughout the one-year study period, dingoes were detected across the landscape at all 23 camera trap stations. Thirty-six out of 66 marked dingoes were sighted at multiple camera stations (i.e., moving dingoes), spanning up to four camera stations (Figures S1 and S2). Dingoes were observed travelling individually or in groups of two to five individuals.

The half-Normal function was selected as it produced a lower AIC_c_ value compared to the hazard half-Normal function on the null model (Table S1). The null half-Normal model with adjustment of effort defined as the number of operational days (i.e., all cameras within a station treated as fully dependent) provided the best fit and, consequently, this type of effort adjustment was used for all subsequent models. While allowing density to vary spatially and temporally, the camera trap data were best fitted (according to lowest AIC_c_ values) by the model incorporating the following detection covariates in combination: (1) a variation of *g0* between the two types of stations (road-based versus focal point stations) and (2) a variation of sigma across sessions. By including the two selected detection covariates, the model with density varying only by season provided the best fit and was therefore chosen as the final model. This model indicated that dingoes were present at a higher density (0.147 dingoes per km^2^) during the wet season compared to the dry season (0.135 dingoes per km^2^) (Table 1), across the habitat mask which covered an area of 613 km^2^. The baseline encounter probability (*g0*) was almost 3 times higher in road-based stations compared to focal point stations. The estimates of the spatial scale parameter (sigma) and the corresponding 95% bivariate Normal home range size were as low as 0.65 km and 8.0 km^2^, respectively, during session 4 and ranged up to 1.25 km and 29.4 km^2^, respectively, during session 2. Given the scarcity of the unambiguous identifications-only dataset, which resulted in failure of multiple models to converge, the entire model selection process was not conducted. Instead, this subset of data was fitted to the final selected model, which generated results consistent with those from the model using the entire dataset, with only 6%–12% differences in density estimates and 2%–15% in the scale parameter estimates. In accordance with the model using the entire dataset, the density estimates from the subset model also increased from the dry season (0.120 dingoes/km^2^; SE = 0.006) to the wet season (0.155 dingoes/km^2^; SE = 0.013), with a lower baseline probability in focal point stations and higher spatial parameter estimates during the dry season compared to the wet season.

**Figure 2:**
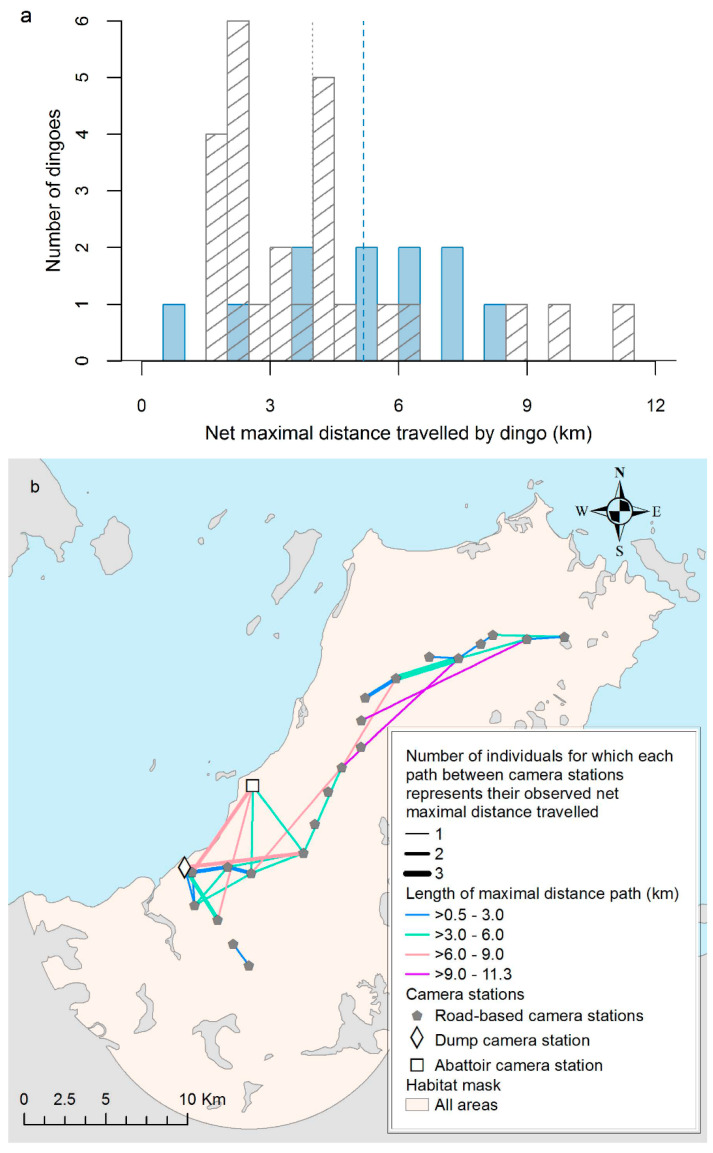
(**a**) Histogram and (**b**) spatial distribution of the paths representing the observed net maximal distance travelled for each marked individual moving dingo (n = 36 dingoes) between the two camera stations furthest apart per individual, based on a camera trap study conducted in the Northern Peninsula Area (NPA) of Queensland, Australia, from May 2016 to May 2017. In (**a**), the two types of paths were compared: paths between a focal point station and a road-based station (blue bars) or between two road-based stations (diagonal gray bars). The mean net maximal distance is shown with the dashed blue line for focal–road paths and the gray dotted line for road–road paths. The connections in (**b**) were weighted according to the number of individuals for which each path represented their net maximal movement observed.

The net maximal distances for the 36 moving dingoes ranged from 0.6 to 8.5 km for focal–road paths, and 1.5 to 11.3 km for road–road paths, but their mean estimates did not differ significantly (*p* = 0.19, Welch *t*-test: Figure 2a). The map of the net maximal distance travelled per individual (Figure 2b) suggests that stations are highly interconnected throughout the study area, with up to three dingoes sharing the same net maximal paths. Although almost one-third of the moving dingoes (n = 11) had their net maximal distance path start or finish at one of the focal points, these observed paths always remained localised around the communities and did not extend further away into the bush area.

**Figure 3:**
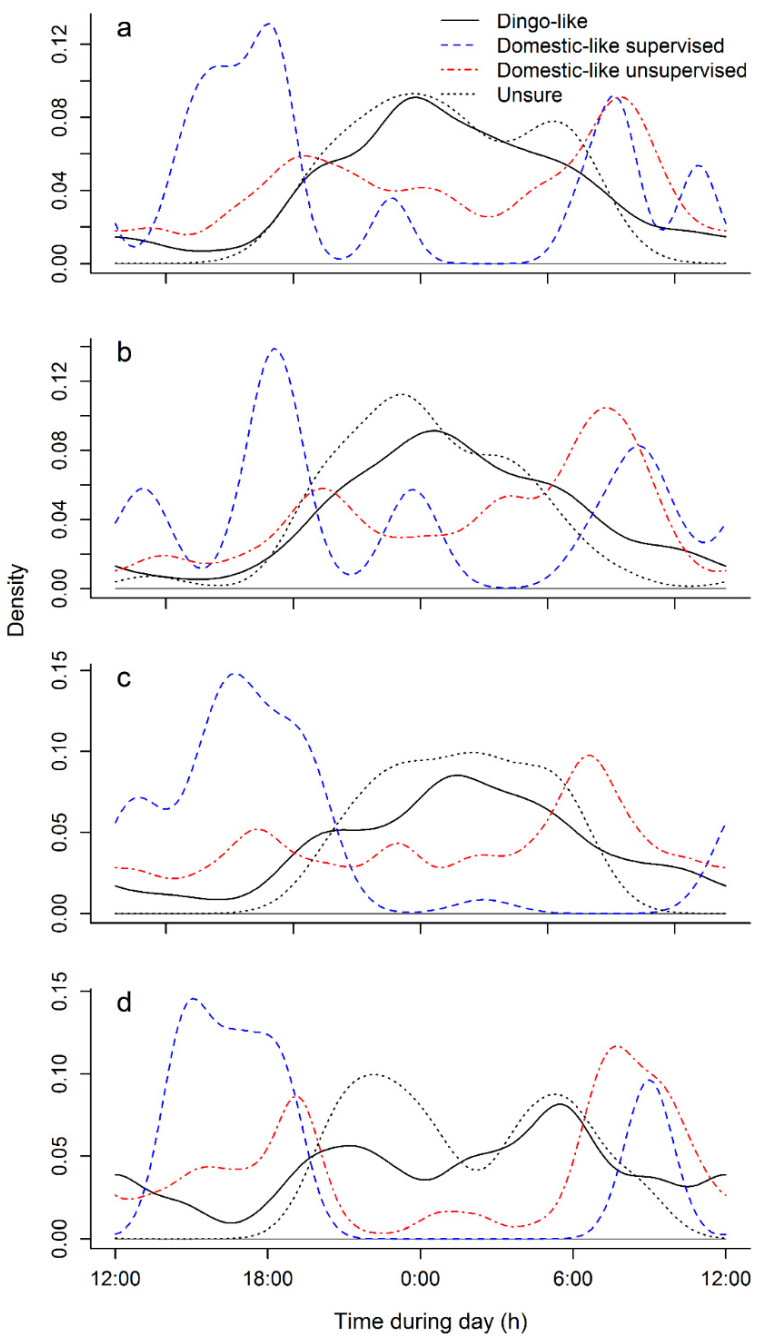
Density curves illustrating the daily activity patterns of dingo-like, domestic-like supervised, domestic-like unsupervised and unsure dogs, based on a camera trap study conducted in the NPA of Queensland, Australia, for (**a**) session 1 (9-May-2016 to 18-Aug2016), (**b**) session 2 (19-Aug-2016 to 16-Nov-2016), (**c**) session 3 (17-Nov-2016 to 14-Feb-2017) and (**d**) session 4 (15-Feb-2017 to 15-May-2017).

Dingoes were predominantly nocturnal, active from sunset to sunrise throughout the year for all four sessions (Figure 3). In general, high peaks of activity were noticeable in the afternoon and early morning for supervised domestic dogs, whereas the activity remained at relatively higher levels throughout the night and morning for their unsupervised counterparts—with the exception of session 4, which showed very little activity at night. Unsupervised domestic dogs exhibited highly overlapping activity times with dingoes during all four sessions, occurring predominantly during nocturnal hours, whereas supervised domestic dogs had lower temporal overlap with dingoes (Table 2). Furthermore, spatial use was positively correlated between dingoes and domestic dogs for all four sessions. However, the only statistically significant spatial correlation (*p* < 0.5), which also showed the highest strength of association, was found between dingoes and domestic unsupervised dogs during session 1.

## 4. Discussion

This is the first study to report the use of a spatially explicit density estimation approach for dingoes in Australia and the first to provide density estimates for dingoes inhabiting an equatorial climate zone of northern Australia. Our results, which ranged between 0.135 dingoes/km^2^ in the dry season and 0.147 dingoes/km^2^ in the wet season, were intermediate within the reported dingo density estimates throughout different habitat types and climate zones in Australia (0.01–0.7 individuals/km^2^) [13]. Our seasonal densities were consistent with that estimated in Kakadu National Park in the Northern Territory of Australia (0.14 dingoes/km^2^) [56], which is characterised by a tropical climate zone and comprises a mixture of forests and wetlands, and comparable to our study area. Our results also fall within the middle of the range of considered density values parametrised in the recently developed rabies spread model of dingoes in northern Australia (0.05–0.38 dingoes/km^2^) [22]. Since specific unsuitable habitat types for dingoes were excluded from the habitat mask, it is important to note that the density values reported in this study correspond to habitat-specific density estimates.

As observed in the model selection table, the differences in AIC_c_ values for the models from the last step of the model selection process (selection of density covariates) were small, especially for the second top model which produced ΔAIC_c_ of less than 2 units. This competing model could also explain our data adequately [57], but the selection of the final model had an insignificant impact on our estimates. The second top model generated a density estimate corresponding to the average value between the two seasonal density estimates from the top model, with only a 0.8%–3% difference in the baseline encounter probability and scale parameter estimates (Table S2). Furthermore, the density model with seasonal variation, which was selected objectively based on our model selection criteria, is biologically plausible. In fact, although the difference in density estimates between seasons is small, the seasonal effect observed in our model (lower dingo population during the dry season compared to the wet season) is most likely associated with the introduction of young dingoes into the population. According to a few observational studies from tropical and subtropical Queensland, the birth period occurs approximately from June to August, followed by a period of rearing and nurturing of pups between July and September [58,59]. During this time, the young pups have a very small chance of detection in camera traps because their activity is limited to close proximity to the den. Young dingoes then become independent, increasing their roaming behaviors in late November [58,60], which corresponds to the beginning of the wet season. Consistent with the literature, all young marked dingoes were first detected between the months of November and January in our camera trap study. From an epidemiologic perspective, for diseases that are transmitted by contact such as rabies, the role of young pups in disease transmission would most likely remain negligible until they reach independence and consequently experience an increased risk of encountering neighboring dingoes. Alternatively, our seasonal densities might be related to a decline in water resources or prey availability during the dry season, resulting in a higher mortality rate in the population. Although dingoes have the ability to shift prey selection depending on relative prey availability as they employ opportunistic foraging strategies [15,61], declining dingo abundance has been correlated with declining prey species [62].

Although the method used in our study to calculate the home range is approximate, our 95% bivariate home range size estimates derived from the SEMR model are consistent with home range estimates reported from the literature in subtropical or tropical climate zones, which vary between 17 and 39.7 km^2^ for 95% contour methods of calculation [13]. We expected the home range size to increase during the mating period and decrease during the nursing period, corresponding to session 4 and session 2, respectively [20,58,59]. However, we estimated a lower spatial scale and home range in session 4, during the second half of the wet season; this could reflect an increased abundance of resource availability throughout the environment, which would diminish the necessity for dingoes to roam long distances to meet their water and dietary requirements. The opposite scenario would apply for session 2, at the end of the dry season, when dingoes would increase their home range area due to scarcity of quality food and water resources. The apparent temporal variations in home range sizes in our context are most likely the result of a complex mixture of multiple factors, with food resources outweighing reproductive behavior because of the dramatic climate variations between seasons. Considering that there seems to be a higher density of dingoes in the NPA during the wet season, with larger home range areas at the end of the dry season, disease transmission could be greater around November (the transition period between the dry season and the wet season).

The largest net maximal distance observed from the focal–road paths (8.5 km) and road–road paths (11.3 km) remained within the normal limits of dingo movements reported in the literature for urban dingoes [35,63]. Additionally, since there was no net maximal path observed between the focal points and stations located further away (i.e., toward the tip of the peninsula, Figure 2b), this suggests that dingoes living in distant areas do not travel long distances to profit from these food resources. According to a conceptual model proposed by Newsome et al. [14], dingoes living near an abundant resource will allow distant neighboring dingoes to enter their home range area transiently to access a shared food supply only if excess food resources are available. The present results might be an indication that food resources in the focal points of the NPA are not sufficient to support this scenario or that resources away from the communities are sufficient to sustain those populations. For instance, one small tourist establishment, which includes a restaurant, is found in the northern part of the peninsula and may contribute in providing localised food supply for dingoes in this area, although we believe these resources would be limited. GPS collaring studies such as Newsome et al. [36] might elucidate such wider-range movements to focal anthropogenic resources.

Unlike Newsome et al. [14], who proposed that resource richness can favour larger group sizes and smaller home range sizes, outcomes from our model did not provide evidence that proximity to NPA communities would have an important influence on either dingo density or home range size. Our results concord with Morrant [35], who did not observe smaller home range sizes for dingoes living in close proximity to human settlements with access to anthropogenic food. Dingo populations near the NPA communities might not rely predominantly on the resources found within or near these communities. Alternatively, the spatial effect of human resources on dingo density or movement might be present, but at a larger scale than our study area.

A higher level of activity of dingoes during crepuscular and nocturnal hours has been documented previously in various studies across Australia [64,65,66], although the tendency is not always consistent, since activities predominantly occurring during daylight hours have also been observed [35]. Contrary to the findings of Sparkes et al. [65] in southeastern Australia, the substantial amount of temporal overlap between dingoes and unsupervised domestic dogs observed within our study, as well as their strong correlation in spatial use during the first half of the dry season, suggests that the common practice in the NPA of allowing community dogs to free-roam might increase the risk of transmission of shared pathogens, namely canine rabies, at the wild–domestic interface. As expected, domestic dogs accompanied by humans were more active during daylight hours, thereby avoiding temporal overlap with dingoes. However, during the dry season, a small peak of activity in supervised dogs was noticeable at night, when dingoes are also most active. This nighttime observation is most likely related to pig hunting activities with the assistance of domestic dogs, a common practice embedded into the traditions of the NPA Indigenous communities. Hunting trips in the NPA occur mainly in the bush, occasionally at night, and can result in encounters with dingoes as reported by hunters [67]. Furthermore, hunting dogs often roam out of the hunter’s sight, thus becoming unsupervised at one point and increasing the risk of interaction with wild animals. This human-mediated movement of community dogs can therefore create opportunities for disease transmission between owned dogs and dingoes inhabiting areas far away from the communities [68].

The camera trap layout in the present study was limited by financial and logistical constraints. The wilderness of our study area, characterised by densely tropical forested landscape, limited our ability to deploy cameras away from the main road, namely in the northern section of the study area. Consequently, the spatial distribution of the camera traps, which broadly followed a south-west to north-east direction, may have introduced potential bias in our density estimates, especially if the dingos’ home range areas in the NPA are not circular but rather elongated perpendicularly (overestimation) or parallel (underestimation) to the array orientation [69]. Anisotropic models, which are effective at reducing bias when home ranges are oriented and elongated uniformly, are not suitable in the case of a linear array [69] and were therefore not considered in our study. Nonetheless, considering that the spatial scale parameter estimates resulting from the model are relatively small compared to the extent of the study area and that numerous paths and trails are found across the study area facilitating movement of dingoes in any given direction through the dense vegetation, we believe that there would not be one predilection in direction of movement and the assumption of a circular home range area is appropriate. Additionally, according to a field camera trap study comparing several survey designs [26], transect arrays have provided precise density estimates using SEMR models, especially when resighting rates of marked animals were sufficiently high, which is most likely the case in the present study (882 marked detection events over 932).

Although camera placement should maximise detections of the target population [26], the deployment of cameras along roads and paths may have introduced potential biased estimates on the spatial scale parameter, and hence density, if the use of these trail systems were preferentially used by only a subgroup of the population, for instance males as reported in jaguars [70,71]. However, multiple GPS studies in the literature have not found statistical differences between male and female dingo home range sizes [18,63,72], suggesting that such a bias is most likely not a concern for our target population.

The SEMR models rely on the assumption of independence among individuals, in terms of their detection and home range centroids distribution, which might not be the case for dingoes because of their territorial and social behaviors. However, violation of this assumption in such a manner has been shown not to affect, in most cases, the bias and precision of density and spatial scale parameter estimates if the levels of aggregation (i.e., pack size) and cohesion (i.e., dependency of space use) in the population are low to moderate [73,74]. Dingoes most likely fit into this category, since pack sizes are relatively small, ranging between one and nine individuals in an optimal habitat providing abundant resources [75,76], and dingo members from a pack will often travel or hunt either individually or in small subgroups [20,77]. Considering the low to moderate expected spatial dependency of this species, the probability of false inferences in our study remains relatively low [74].

Perfect and equal detectability across stations and time is impossible despite best efforts, since various underlying factors will compromise detection probabilities. A spatially explicit approach allows the correction of potential detection bias by accounting for heterogeneous detection probabilities. For our SEMR models, we included an adjustment for varying sampling effort through the number of operational days for each station. Furthermore, we accounted for the potential varying detection probabilities across time and stations caused by vegetation growth partially obstructing camera detection zones. However, the inclusion of the latter covariate in the model did not yield a better fit and was therefore most likely not a significant factor influencing detection probability. Other uncontrollable factors might have also resulted in inconsistent detection probabilities. For example, the passive infrared heat-in-motion sensors used in this study are unable to detect an animal in movement if the ambient temperature is equivalent to the body temperature of the animal [78]. This phenomenon might partially explain the lower baseline probability of detection, *g0*, in focal point stations, despite the larger number of camera traps within those stations. Cameras at the focal point stations were located in areas without shelter, in direct sunlight, compared to cameras from the road-based stations, which were mostly located in areas covered by vegetation and trees. In fact, the average ambient temperature captured from the camera traps was higher in the focal points stations (34.1 °C) compared to the road-based stations (32.0 °C), which could consequently affect animal detection. The differences in the baseline probability of detection might also indicate that dingoes avoid areas characterised by occasional human activity, irrespective of the presence of abundant food resources.

Errors of discrimination between the two contrasting populations of dingo-like (i.e., wild dogs) and domestic-like unsupervised dogs (i.e., community dogs) could have occurred, leading potentially to an impact on the SEMR analyses as well as the temporal activity curves. With an expanding population of dingo–dog hybrids throughout Australia, the classification of dingoes, hybrids and domestic dogs based solely on phenotypical characteristics, without the recourse of genetic testing, reduces discriminatory capacity of observers [79]. Nevertheless, most pictures in this study provided sufficient information to categorise the animals confidently. Similarly, by relying on identification of natural marks using camera trap data in our study, misidentifications of individual dingoes cannot be fully excluded, which might lead to biased density estimates [80]. However, considerable efforts were taken to reduce the likelihood of misidentifying dingoes by obtaining consensus of all identifications through mutual consultations between two researchers. We believe this process increased the robustness of our approach and maximised confidence in our identifications. Furthermore, the strong convergence of estimates between the models using the two levels of confidence for identifications strengthens the validity of our data and indicates that errors due to misidentifications would not substantially bias the overall conclusions.

Although camera trap studies can provide useful information on movement characteristics, activity patterns and potential home range sizes, the data generated for this specific purpose are limited compared to other methods such as the use of GPS collars in telemetry studies. There is a substantial amount of unobserved data from camera trap studies, which makes it difficult to draw unequivocal conclusions. The landscape within the study area was not surveyed in its entirety (because this is physically impossible). Consequently, the observed movements represent only a small subsample of the actual routes travelled by NPA dingoes throughout the study period, especially in the north where the layout of the cameras was not arranged as a two dimensional array therefore limiting detection of movement to one direction only. Despite these limitations, the use of a camera trap sampling design has enabled the monitoring of dingo movements of a large number of individuals as an alternative to telemetry, providing preliminary insights into movement patterns of dingoes in the NPA.

## 5. Conclusions

Our study establishes a foundation for density estimates of dingoes in the NPA in northern Queensland, where there is a risk of rabies incursion. Furthermore, the results can provide insight into possible densities of dingoes in areas elsewhere in northern Australia. Densities and home range sizes fluctuated temporally across seasons, and this tendency might apply to areas with similar strong and regular climatic seasonality. Overall, our data suggest that dingoes are spatially comparable throughout the NPA with respect to density, home range and movement patterns regardless of the distance to human-provided food resources in communities. This indicates that a relatively simple and parsimonious disease model, without the incorporation of varying ecological parameters between dingoes from an anthropogenic area compared to a natural and remote environment, might be sufficient to reflect spatial disease transmission within the dingo population in the NPA.

## Figures and Tables

**Table 1:** Parameter estimates (density, sigma and *g0*) and derived estimates (95% bivariate home range size) from the final model of a spatially explicit multi-session mark–resight model, using a half-Normal detection function, for a dingo population in the NPA of Queensland, Australia, monitored from May 2016 to May 2017 (Session 1: May 9th–Aug 18th; Session 2: Aug 19th–Nov 16th; Session 3: Nov 17th–Feb 14th; Session 4: Feb 15th–May 15th).

Estimates	Mean		(95% CI)
Parameter estimates of model			
Density (individuals per km^2^)			
Dry (Session 1 and 2)		0.135	(0.127–0.144)
Wet (Session 3 and 4)		0.147	(0.135–0.159)
Sigma (km)			
Session 1		1.16	(1.10–1.23)
Session 2		1.25	(1.17–1.34)
Session 3		1.07	(0.99–1.15)
Session 4		0.65	(0.59–0.71)
*g0*			
Focal point stations		0.131	(0.108–0.159)
Road-based stations		0.376	(0.314–0.442)
Derived estimates from model			
95% home range size (km^2^)			
Session 1		25.3	(22.6–28.4)
Session 2		29.4	(26.0–33.8)
Session 3		21.5	(18.5–24.9)
Session 4		8.0	(6.4–9.5)

**Table 2:** Temporal overlap of daily activity patterns, and spatial correlation of dingoes and supervised and unsupervised domestic dogs in the NPA of Queensland, Australia, monitored with camera traps from May 2016 to May 2017 (Session 1: May 9th–Aug 18th; Session 2: Aug 19th–Nov16th; Session 3: Nov 17th–Feb 14th; Session 4: Feb 15th–May 15th).

Session	Dingo/Supervised Domestic Dog				Dingo/Unsupervised Domestic Dog			
	Temporal		Spatial		Temporal		Spatial	
	Overlap Estimate	(95% CI)	Spearman Coefficient	(95% CI)	Overlap Estimate	(95% CI)	Spearman Coefficient	(95% CI)
Session 1	0.34	(0.27–0.41)	0.10	(−0.33–0.49)	0.70	(0.65–0.75)	0.63 *	(0.28–0.82)
Session 2	1.41	(0.26–0.57)	0.39	(−0.08–0.72)	0.69	(0.62–0.75)	0.41	(−0.05–0.73)
Session 3	0.25	(0.17–0.33)	0.17	(−0.32–0.59)	0.69	(0.64–0.75)	0.43	(−0.05–0.75)
Session 4	0.26	(0.10–0.42)	0.12	(−0.42–0.60)	0.55	(0.47–0.64)	0.28	(−0.27–0.69)

* *p*-value < 0.05.

## Supplementary Materials

The following are available online at https://www.mdpi.com/2076-2615/10/5/865/s1, Figure S1: Number of movements between camera stations for each moving marked individual dingo (n = 36), based on captures from a camera trap study conducted in the NPA of Queensland, Australia, from May 2016 to May 2017; Figure S2: Number of stationary dingoes per station (n = 30), based on captures from a camera trap study conducted in the NPA of Queensland, Australia, from May 2016 to May 2017; Table S1: AIC_c_ values from each SEMR multi-session model considered in the model selection process, based on a camera trap study to estimate density of a dingo population in the NPA of Queensland, Australia, monitored from May 2016 to May 2017; Table S2: Parameter estimates (density, sigma and g0) from the second top model of a spatially explicit multi-session mark–resight model, using a half-Normal detection function, for a dingo population in the NPA of Queensland, Australia, monitored from May 2016 to May 2017 (Session 1: May 9th–Aug. 18th; Session 2: Aug. 19th–Nov. 16th; Session 3: Nov. 17th–Feb.14th; Session 4: Feb. 15th–May 15th).
