# Supplementary material for: Dingo Density Estimates and Movements in Equatorial Australia: Spatially Explicit Mark–Resight Models
Source: Animals (Basel). 2020 May 17;10(5):865. doi: 10.3390/ani10050865 (PMC7278439; doi:10.3390/ani10050865)
Supplement: Supplementary file 1 [file animals-10-00865-s001.pdf]

Dingo density estimates and movements in equatorial Australia: Spatially explicit mark-resight models

Vanessa Gabriele-Rivet*, Julie Arsenault, Victoria Brookes, Peter J.S. Fleming, Charlotte Nury & Michael P. Ward

Correspondence: Vanessa Gabriele-Rivet vgab5928@uni.sydney.edu.au

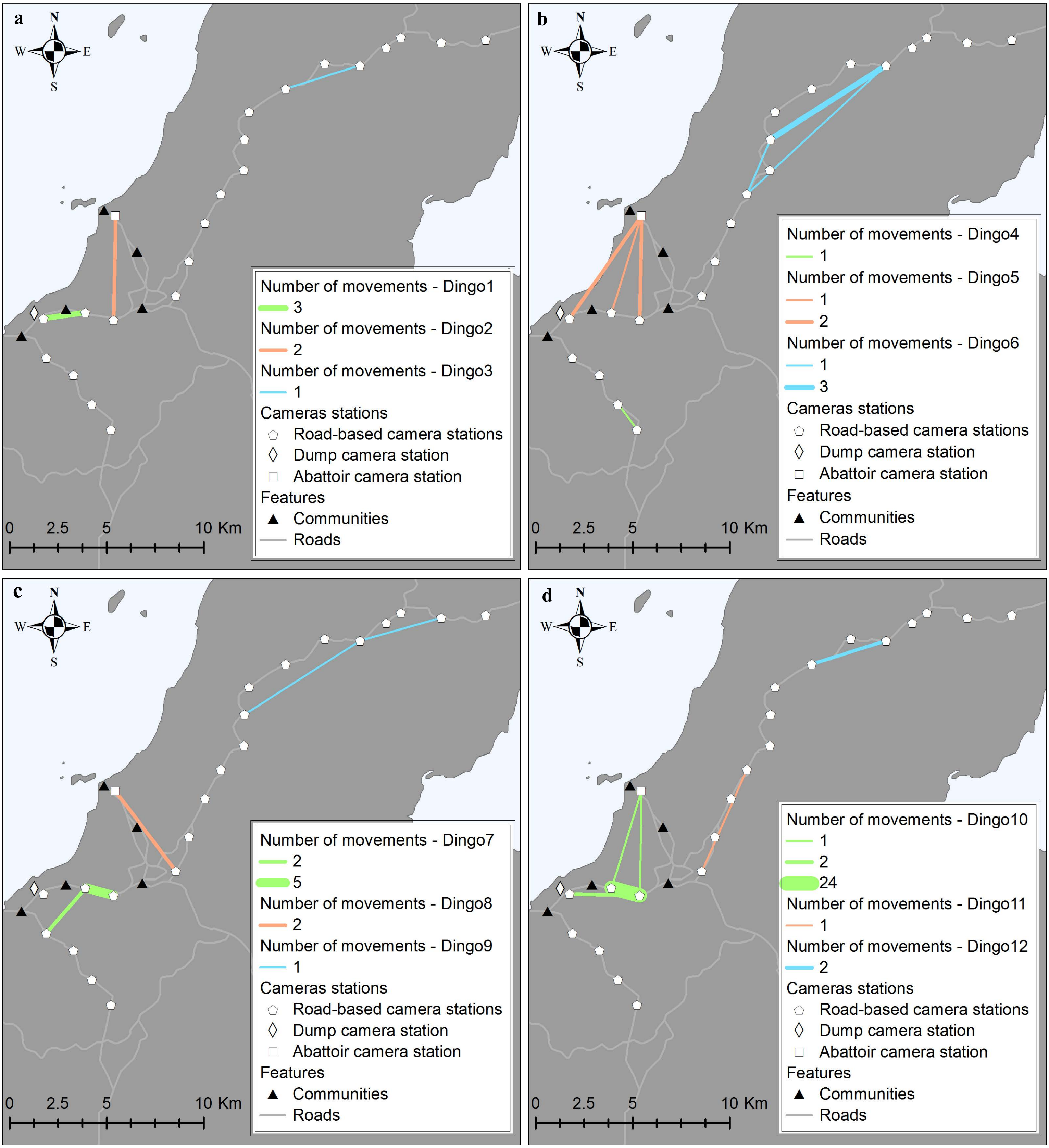

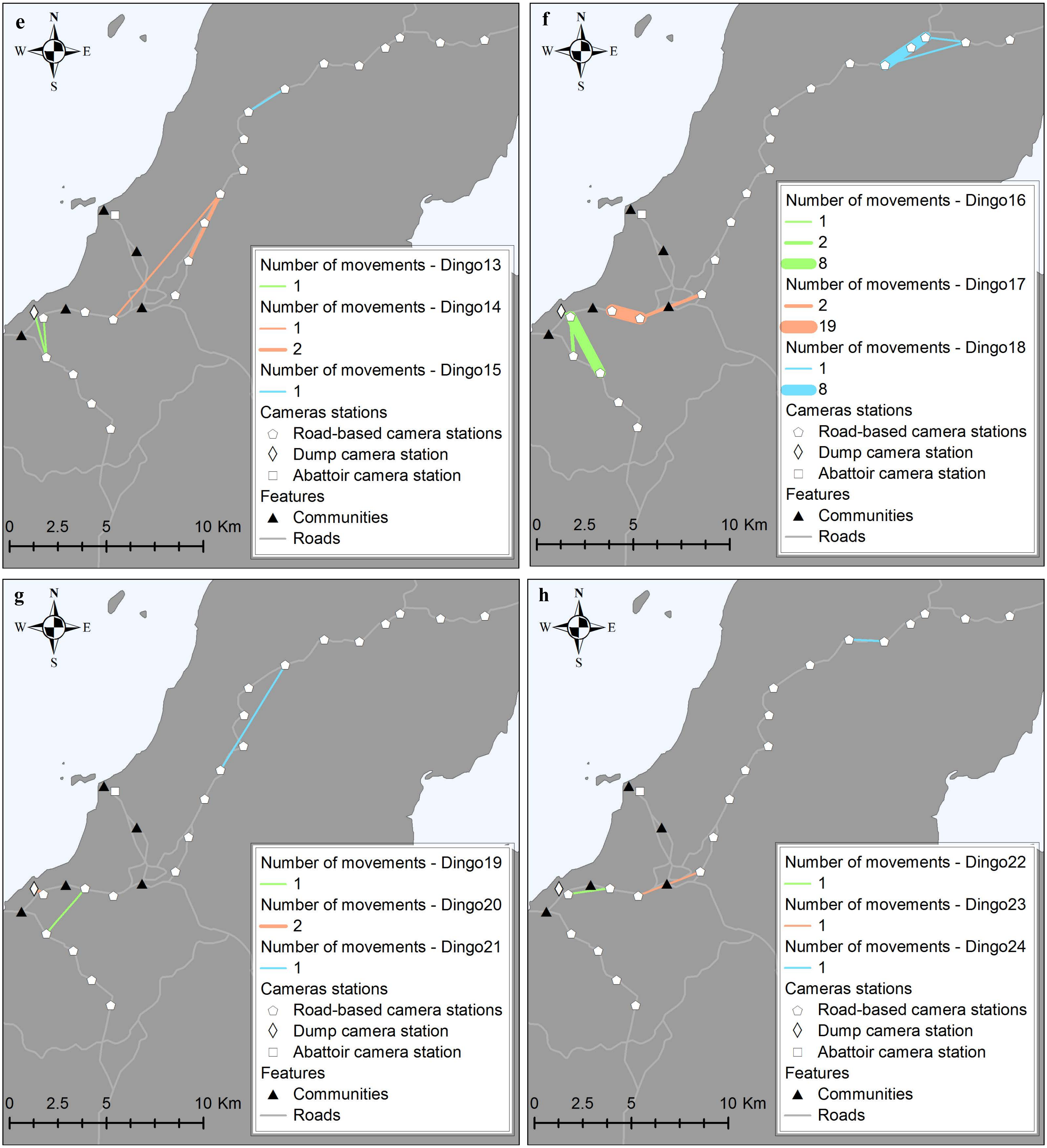
e
f
g
h
N
S
E
W
Number of movements - Dingo13
1
Number of movements - Dingo14
1
2
Number of movements - Dingo15
1
Number of movements - Dingo16
1
2
8
Number of movements - Dingo17
2
19
Number of movements - Dingo18
1
8
Number of movements - Dingo19
1
Number of movements - Dingo20
2
Number of movements - Dingo21
1
Number of movements - Dingo22
1
Number of movements - Dingo23
1
Number of movements - Dingo24
1
Cameras stations
Road-based camera stations
Dump camera station
Abattoir camera station
Features
Communities
Roads
0
2.5
5
10 Km

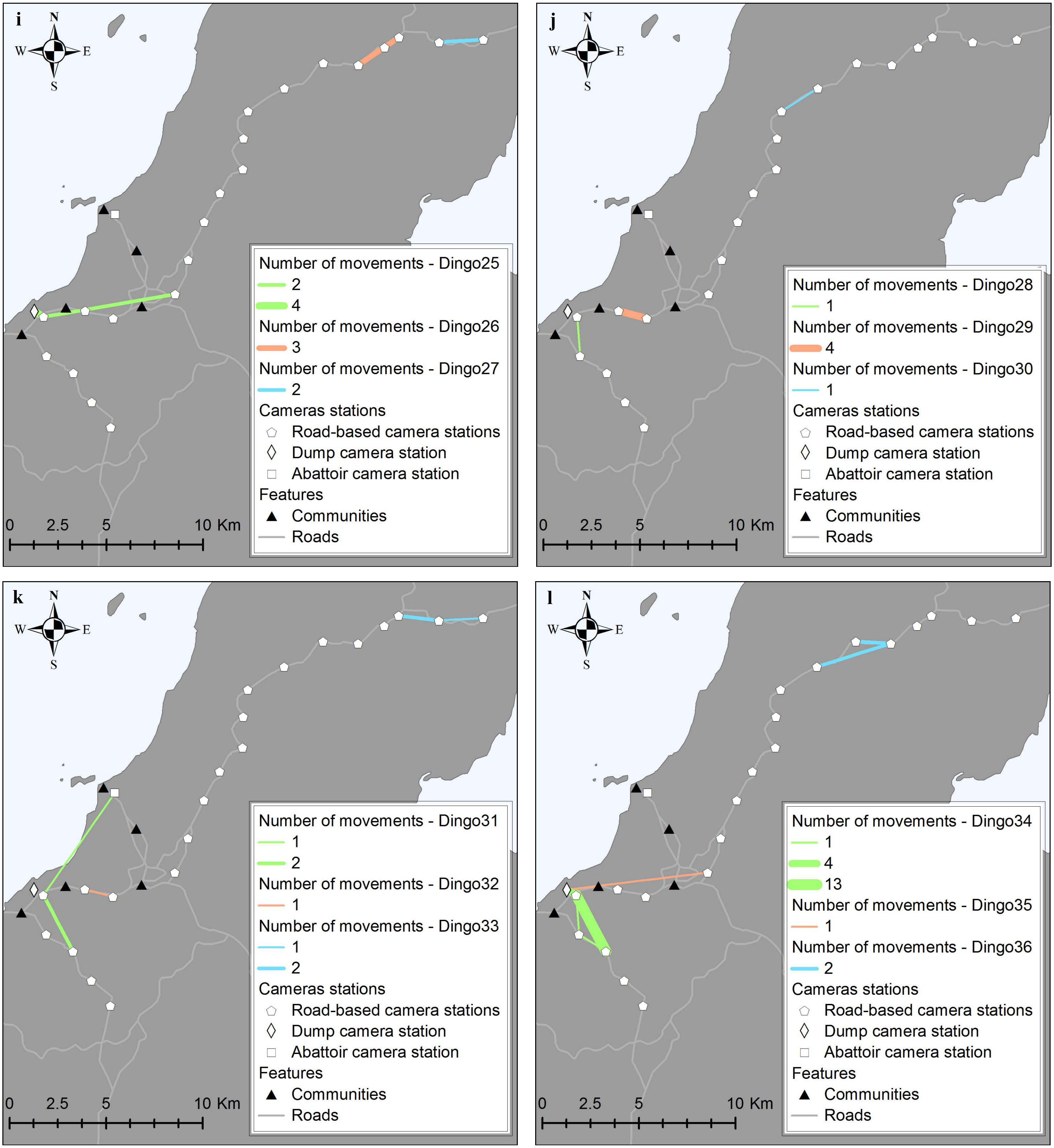

Fig. S1 (a-l) Number of movements between camera stations for each moving marked individual dingo (n=36), based on captures from a camera-trap study conducted in the NPA of Queensland, Australia, from May 2016 to May 2017

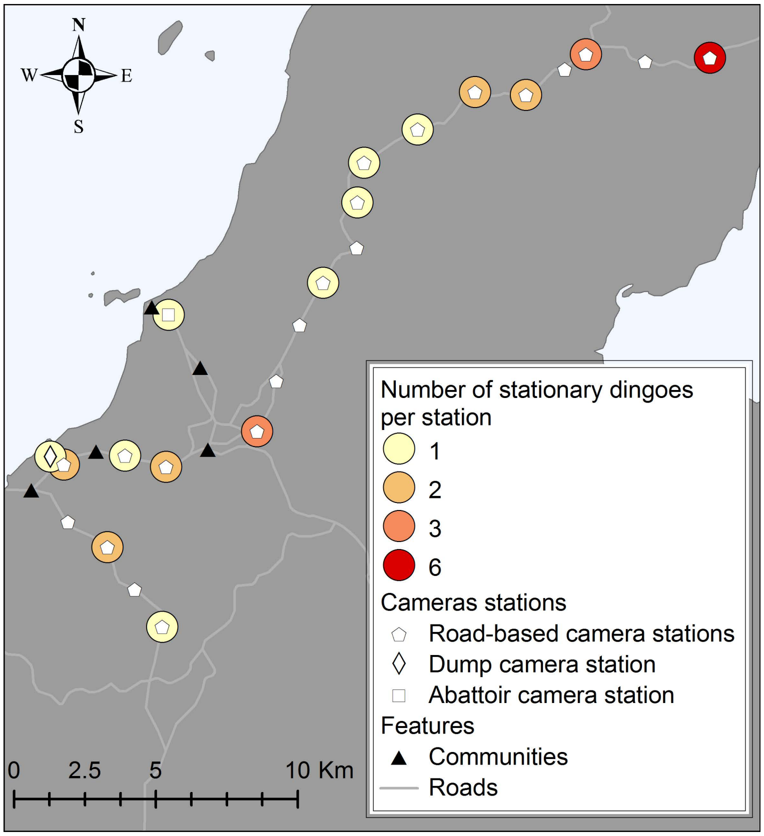

Fig. S2 Number of stationary dingoes per station (n=30), based on captures from a camera-trap study conducted in the NPA of Queensland, Australia, from May 2016 to May 2017

Table S1. SEMR multi-session models ranked based on Akaike Information Criteria corrected for small sample size (AICc), based on a camera-trap study to estimate density of a dingo population in the NPA of Queensland, Australia, monitored from May 2016 to May 2017.

Detection function	Effort adjustment	Density covariates	Detection covariates		Nb param	logLik	AIC_c	ΔAIC_c	*w*
			g0	sigma					
Step 1									
HN	N	N	N	N	4	-3193.8	6396.0	0	1.00
HHN	N	N	N	N	4	-3253.6	6515.5	119.5	0.00
Step 2									
HN	Effort 2	N	N	N	4	-3171.0	6350.2	0	1.00
HN	N	N	N	N	4	-3193.8	6396.0	45.8	0.00
HN	Effort 1	N	N	N	4	-3210.1	6428.4	78.2	0.00
Step 3									
HN	Effort 2	Habitat + Session	Site	Session	12	-3097.6	6222.0	0	1.00
HN	Effort 2	Habitat + Session	Site	N	9	-3114.7	6248.9	29.6	0.00
HN	Effort 2	Habitat + Session	N	Session	11	-3134.7	6293.6	71.6	0.00
HN	Effort 2	Habitat + Session	Nb cams	N	9	-3138.9	6297.3	75.4	0.00
HN	Effort 2	Habitat + Session	N	Season	9	-3140.3	6300.1	78.1	0.00
HN	Effort 2	Habitat + Session	Visibility	N	9	-3147.6	6314.7	92.7	0.00
HN	Effort 2	Habitat + Session	N	N	8	-3150.8	6318.8	96.8	0.00
HN	Effort 2	Habitat + Session	N	Habitat	9	-3150.7	6320.9	98.9	0.00
Step 4									
HN	Effort 2	Season	Site	Session	9	-3098.1	6215.7	0	0.40
HN	Effort 2	N	Site	Session	8	-3099.5	6216.3	0.6	0.30
HN	Effort 2	Habitat + Season	Site	Session	10	-3098.1	6218.1	2.4	0.12
HN	Effort 2	Habitat	Site	Session	9	-3099.5	6218.5	2.8	0.10
HN	Effort 2	Session	Site	Session	11	-3097.7	6219.6	3.9	0.06
HN	Effort 2	Habitat + Session	Site	Session	12	-3097.6	6222.0	6.3	0.02

Nb param = Number of parameters; logLik = Log likelihood value; w = weight of each model; HN = Half-Normal detection function; HHN = Hazard Half-Normal detection function; Effort 1 = Number of camera trap days; Effort 2 = ; Number of operational days; Habitat = 2-class spatial environment covariate; Site = 2-class station-specific covariate allowing the distinction of road-based from focal point stations; Session = Categorical session-specific covariate to explore differences between all 4 sessions; Season = 2-class season-specific covariate to explore differences between the dry season (sessions 1 and 2) and the wet season (sessions 3 and 4); Nb cams = Continuous station-specific covariate comprising the average number of active cameras, based on days with at least one active camera, for each station; Visibility = Continuous degree of visibility covariate, taking into account obstruction due to vegetation growth at each station and each occasion.

Table S2. Parameter estimates (density, sigma and g0) from the second top model of a spatially explicit multi-session mark–resight model, using a half-Normal detection function, for a dingo population in the NPA of Queensland, Australia, monitored from May 2016 to May 2017 (Session 1: May 9th – August 18th; Session 2: August 19th – November 16th; Session 3: November 17th – February 14th; Session 4: February 15th – May 15th).

	**Mean**	**(95 % CI)**
Parameter estimates of model		
Density (individuals per km^2)	0.141	(0.132-0.151)
Sigma (km)		
Session 1	1.14	(1.08-1.21)
Session 2	1.24	(1.15-1.34)
Session 3	1.11	(01.03-1.18)
Session 4	0.66	(0.60-0.72)
g0		
Focal point stations	0.132	(0.109-0.160)
Road-based stations	0.366	(0.311-0.423)
